# Antennal sensory structures of *Phenacoccus solenopsis* (Hemiptera: Pseudococcidae)

**DOI:** 10.1186/s12983-022-00479-4

**Published:** 2022-12-15

**Authors:** Nesreen M. Abd El-Ghany, Jing-Jiang Zhou, Youssef Dewer

**Affiliations:** 1 Pests and Plant Protection Department, Agricultural and Biological Research Institute, National Research Centre, 33 El-Buhouth Street, Dokki, Giza, 12622 Egypt; 2 State Key Laboratory of Green Pesticide and Agricultural Bioengineering, Biocontrol Engineering Laboratory of Crop Diseases and Pests of Gansu Province, No. 1 Yingmen Village, Anning District, Lanzhou, 730070 China; 3 Phytotoxicity Research Department, Central Agricultural Pesticide Laboratory, Agricultural Research Center, 7 Nadi El-Seid Street, Dokki, Giza, 12618 Egypt

**Keywords:** Mealybug, *Phenacoccus solenopsis*, Scanning electron microscopy, Antennae, Sensilla, Olfactory receptor

## Abstract

**Background:**

The cotton mealybug *Phenacoccus solenopsis* Tinsley (Hemiptera: Pseudococcidae) is one of the most devastating sap-sucking pests of cultivated plants. The success of *P. solenopsis* is attributable to its ecological resilience and insecticide resistance, making its control extremely difficult and expensive. Thus, alternative safe approaches are needed to prevent the pest population from reaching the economic threshold. One of these novel approaches is based on the fact that chemical communication via the olfactory system drives critical behaviors required for the survival and development of the species. This knowledge can be useful for controlling insect pests using traps based on semiochemicals. The antennae of insects are an invaluable model for studying the fundamentals of odor perception. Several efforts have been made to investigate the histological and ultrastructural organization of the olfactory organs, such as the antennae and maxillary palps, in many insect species. However, studies on the antennal sensory structures of *Phenacoccus* species are lacking. Furthermore, although enormous progress has been made in understanding the antennal structures of many mealybug species, the olfactory sensilla in the antennae of *P. solenopsis* have not yet been described. In this study, we describe, for the first time, the morphology and distribution of the antennal sensilla in male and female *P. solenopsis* using scanning electron microscopy.

**Results:**

Our results revealed that the entire antennae length and the number of flagellar segments were different between the sexes. Eight morphological types of sensilla were identified on male antennae: trichoid sensilla, chaetic sensilla (three subtypes), basiconic sensilla (two subtypes), and campaniform sensilla (two subtypes). Six morphological types of sensilla were found on female antennae. Sensilla chaetica of subtype 2 and campaniform sensilla of subtype 1 were distributed only on male antennae, suggesting that these sensilla are involved in the recognition of female sex pheromones. The subtype 1 of sensilla chaetica was significantly more abundant on female antennae than on male ones, while subtype 3 was only located on the terminal flagellar segment of the antenna in both sexes.

**Conclusions:**

This study provides insightful information for future electrophysiological and behavioral studies on chemical communication in insects, particularly the cotton mealybug, *P. solenopsis* that could help in developing new strategies for controlling this economically important insect species*.*

## Background

Invasive mealybugs (Hemiptera: Pseudococcidae) are soft-bodied scale insects that are widely distributed throughout the world [1, 2]. Based on the systematic catalog compiled by Ben-Dov [3], 1981 species in 290 genera have been described worldwide. Some species are notorious agricultural pests that damage major crops, including cotton, rice, sugarcane, cassava, potato, tomato, and many fruits [4]. The ability of the mealybugs to feed on diverse plants helps them find a suitable host shortly after being introduced to a new region and establishing their populations [5]*.* They destroy plants by directly depleting sap as well as by transmitting plant viruses [6]. Furthermore, they excrete honeydew, which promotes microbial growth and severely reduces crop quality [7]. The genus *Phenacoccus* comprises approximately 180 species and is one of the largest genera in Pseudococcidae [3]. In Egypt, the genus *Phenacoccus* is represented by eight species [4].

The cotton mealybug *Phenacoccus solenopsis* Tinsley (Hemiptera: Pseudococcidae) is one of the most serious polyphagous herbivorous insect pests that can adapt to multiple climates and hosts. Heavy infestations cause direct economic and ecological damages that reduce plant vigor and cause plant death [8]. They attack more than 200 plant species and are found in more than 35 geographical regions worldwide [9]. The success of *P. solenopsis* as a devastating pest on various crops is due to its wide range of morphological traits and ecological adaptability. The infestation intensity of *P. solenopsis* was found to be conversely related to the temperature and negatively correlated with the relative humidity and rainfall [10]. Moreover, the morphological variation between *P. solenopsis, P. solani* Ferris, and *P. defectus* Ferris collected from different parts of the world is likely to reflect the climatic conditions (such as temperature and humidity) experienced by the insects during their development [11]. This invasive species was reported to spread and cause significant economic and environmental damage in 17 provinces and 11 regions of China [12]. In Egypt, *P. solenopsis* was first recorded on weeds by Abd-Rabou et al*.* [13] and, subsequently, as an invasive pest species on various economically important crops, including tomato, cotton, okra, and eggplant, and some ornamental plants [14–16]. More recently, it has become one of the most invasive pests of potatoes in Egypt [4].

The male of *P. solenopsis* is nonfeeding and has a short lifespan of 2–3 days. During this lifespan, the adult male mates with approximately 3–6 females. The males can mated immediately after their emergence [17]. Both nymphs and adult females are plant-sucking feeders that attack different plant parts, causing wilting, stunting, and even death of the whole host plant. The food selection behavior of the female mealybugs has been described by Renard [18]. The first step in feeding process involves the walking on the plant surface, followed by exploratory behavior using the chemo- and mechano-receptors on the antenna and mouthparts. The mealybug antennae are pointed forward, the labium quickly hits the plant surface, and scrapes it with the tips of its legs. Subsequently, mealybug touches the scraped surface with the last flagellar segment of the antenna and the tip of the labium [18]. The mealybug can detect different types of information including (i) information about the nature of the food source via the air above the leaf surface utilizing the olfactory function of its antennae and (ii) information on the chemical and physical nature of the plant surface using its legs, labium, and antenna.

For most insects, the antennae are their primary olfactory sensors [19, 20]. The antennae are equipped with a wide variety of sensillum types. Generally, the sensilla on insect antennae are not randomly distributed [21]. Their pattern may reflect the impact of many interacting selection pressures in which the size of the individual, developmental stages, sex, feeding habits, and habitats are of considerable significance [22]. Early studies have identified overall antennal length and lengths of individual segments as primary characters to discriminate between mealybug species [23, 24]. Calatayud and Le Rü [25] and Le Rü et al. [26, 27] have described the organization of the sensilla on the antennae and the labium in the fourth-instar nymph of the cassava mealybug *P. manihoti* Matile-Ferrero. Other studies have evaluated the external morphology of wax-secreting pores in six economically important invasive mealybug species (Hemiptera: Pseudococcidae) in Sri Lanka: *Coccidohystrix insolita* Green, *Dysmicoccus brevipes* Cockerell, *D. neobrevipes* Beardsley, *Maconellicoccus hirsutus* Green, *P. solenopsis* Tinsley, and *Planococcus lilacinus* Cockerell using scanning electron microscopy (SEM) [27]. Sirisena et al. [28] only reported the number of female antennal segments and setae without identifying the sensillum types. Karam et al*.* [29] provided a morphological description of the antennae of the female mealybug *Ferrisia malvastra* McDaniel. However, the ultrastructural characteristics, sensillum types, and distributions of antennal sensilla of male and female *P. solenopsis* and other soft scales (Pseudococcidae and Coccidae) have been poorly documented. To date, the ultrastructural characteristics and sensillum types of the male antennae have not been reported for any mealybug species. In this study, we investigated the external morphology, sensillum types, and distribution of the olfactory sensilla in the antennae of male and female *P. solenopsis* for the first time using SEM.

To our knowledge, the olfactory receptors on mealybug antennae have not been identified. The mealybug olfactory receptors must be characterized to determine how this pest mates and feeds and understand the mechanisms of chemical communication between males and females prior to mating. Furthermore, morphological evidence on the structure of the male antenna of any mealybug species is lacking. Thus, our study aims to describe and analyze the morphology, structure, distribution, and quantity of sensilla on the antennae of male and female *P. solenopsis.*

## Results

### Male antennae

#### Antennal morphology

**Fig. 1:**
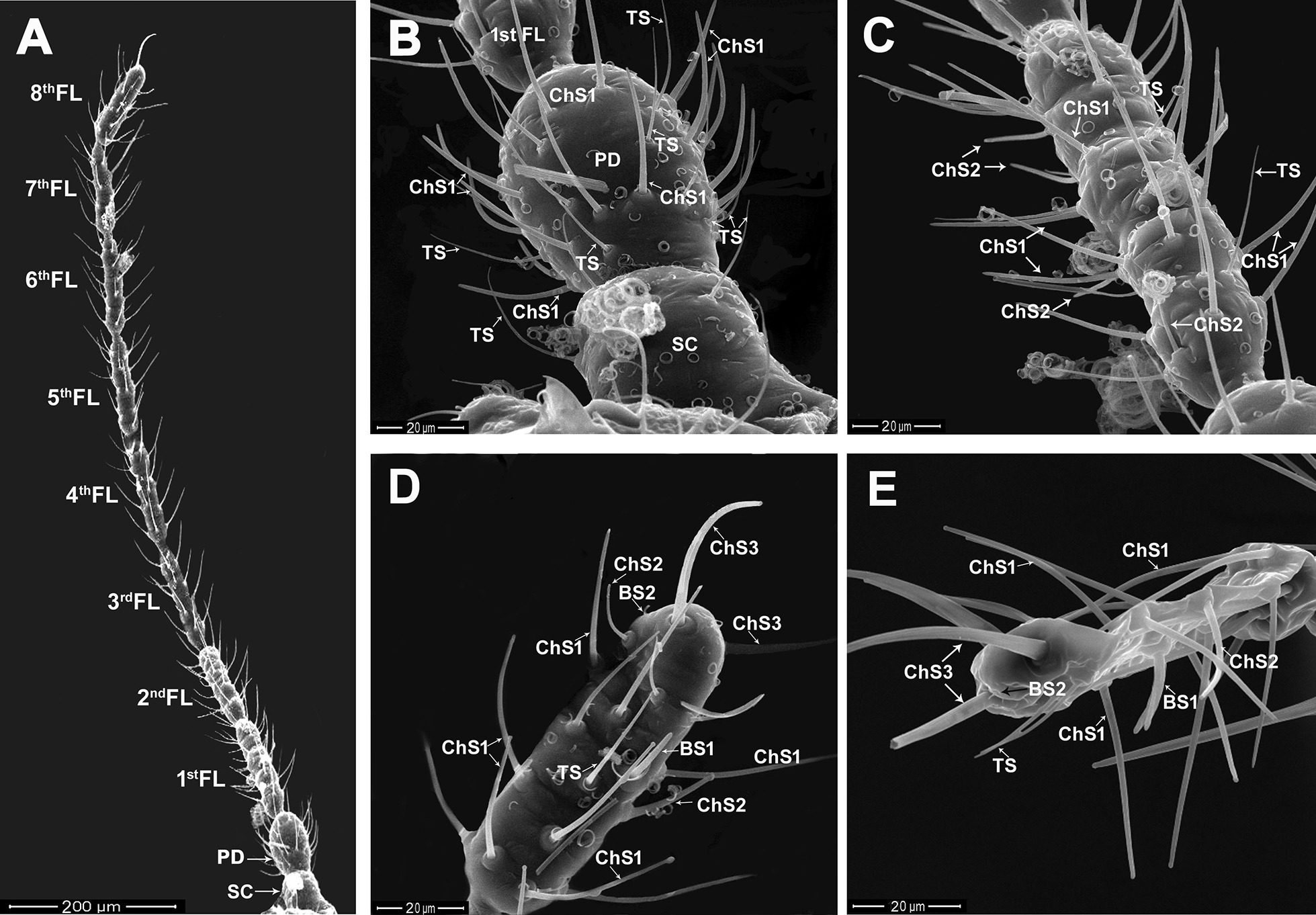
Scanning electron microscopy (SEM) micrographs of the male antenna of *P. solenopsis*. **A** The antennal segments: scape (SC), pedicel (PD), and flagellum consisting of eight flagellomeres (FL). **B** A magnified view of SC, PD, and a part of the first flagellomere. Different sensillum types: trichoid (TS), and chaetica subtype 1 (ChS1) are present. **C** A magnified view of the first flagellomere associated with different sensilla: TS, ChS1, ChS subtype 2. **D** and **E** A magnified view of the terminal flagellar segment (eighth) associated with different sensilla: TS, basiconic sensilla subtype 1(BS1), BS subtype 2, ChS1, ChS2, and ChS subtype 3

The male antenna of *P. solenopsis* is illustrated in Fig. 1A. It consists of ten segments grouped into three regions: scape (Fig. 1B), pedicel (Fig. 1B), and flagellum. The entire antenna length of the adult male is 844.65 ± 7.74 µm, (mean ± S.D.). The scape is the shortest segment of the antenna, roughly squared, with a mean length of 41.18 ± 2.37 μm and a width of 40.57 ± 1.32 μm. The pedicel is the broadest and most elongated antennal segment (45.25 ± 1.86 μm wide) and is longer (55.18 ± 1.84 μm) than the scape segment (Fig. 1B). By contrast, the flagellum (Fig. 1C–E) consists of eight morphologically different rod-shaped sections called flagellomeres. The flagellomeres are the thinnest and longest antennal segments, with a mean length of 92.14 ± 8.58 μm and a width of 24.07 ± 2.23 μm. The smallest segment is the second flagellomere with a mean length of 81.84 ± 1.66 µm and a width of 25.82 ± 1.09 µm. The fourth flagellomere is the longest segment of the flagellum, followed by the third flagellomere segment, with mean lengths of 107.66 ± 1.04 µm and 99.05 ± 1.60 µm and widths of 25.08 ± 0.68 µm and 23.28 ± 1.48 µm, respectively. The thinnest segment is the sixth flagellomere, with a width of 20.70 ± 1.21 µm, followed by the seventh and fifth flagellomeres with widths of 22.20 ± 0.72 µm and 22.40 ± 0.71 µm, respectively. By contrast, the broadest flagellar segment is the terminal flagellaromere (the eighth), followed by the first flagellomere with widths of 26.81 ± 0.85 µm and 26.30 ± 0.99 µm, respectively.

#### Types of sensilla

**Fig. 2:**
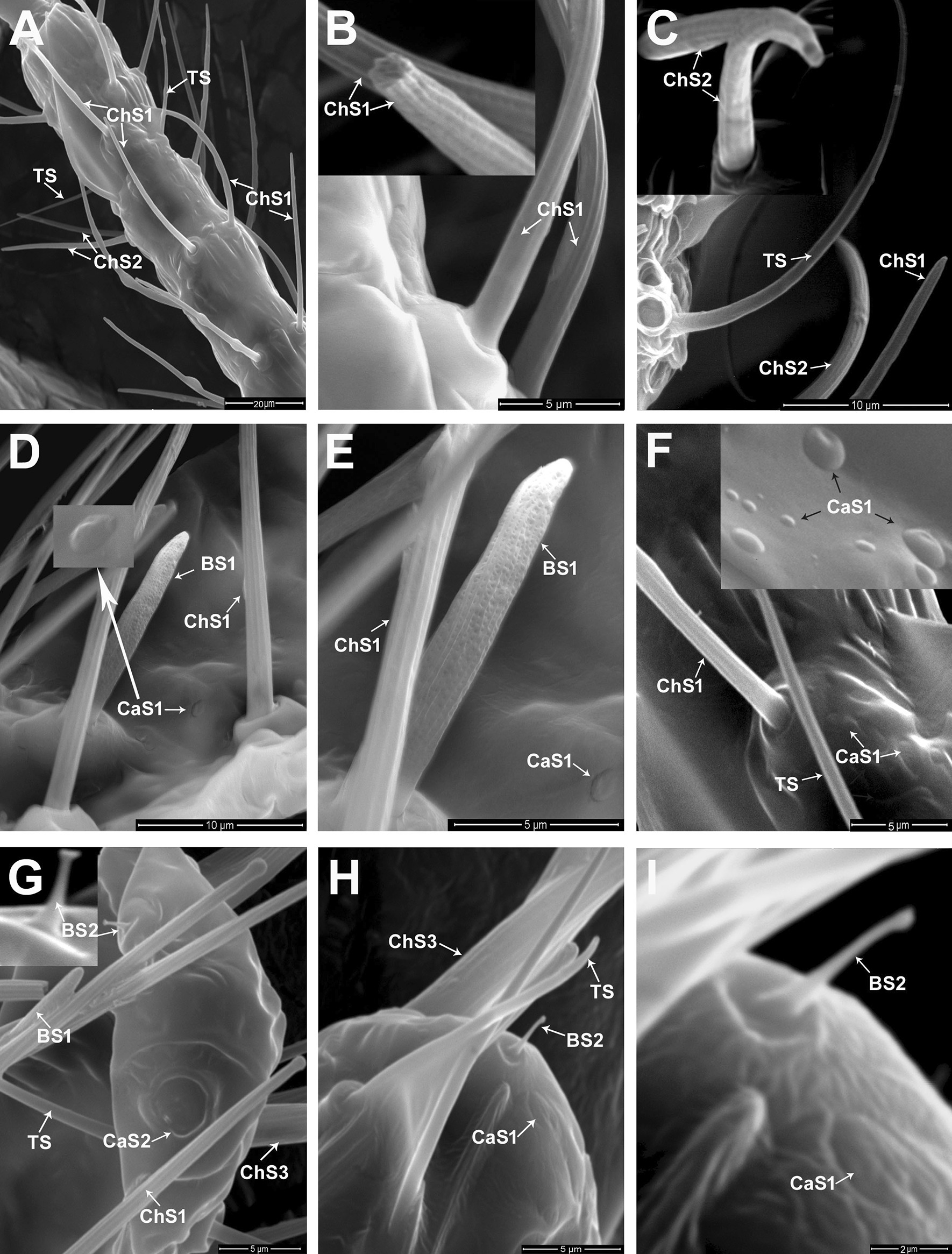
Scanning electron microscopy (SEM) micrographs of the male antenna of *P. solenopsis*. **A** Shows fifth antennal segments with different sensillum types: trichoid (TS) and chaetica subtypes 1 and 2 (ChS1 and ChS2, respectively). **B** A magnified view of chaetica subtype 1 (ChS1). **C** Distribution of different sensillum types on the fifth antennal segment: TS, ChS1, and ChS2; and a higher magnification of ChS2 at the upper left side of the micrograph. **D**, **E** The seventh flagellar segment is associated with different sensillum types: BS subtype 1, ChS subtype 1, and CaS subtype 1 with higher magnification at the upper left side of the D micrograph. **F** The sixth flagellar segment is associated with TS, ChS1, and CaS1 sensilla with higher magnification at the upper right side of the micrograph. **G** The terminal flagellar segment (eighth) associated with different sensillum types: TS, ChS subtype 1, ChS subtype 3, BS subtype 1, BS subtype 2 (higher magnification at the upper left side), and CaS subtype 2. **H** The tip of the terminal flagellomere associated with TS, ChS3, BS2, and CaS1 sensilla. **I** A magnified view of BS2 and CaS1sensilla

Eight sensillum types were identified on different antennal segments: trichoid sensilla (TS), three subtypes of chaetica sensilla (ChS1, ChS2, and ChS3), two subtypes of basiconic sensilla (BS1 and BS2), and two subtypes of campaniform sensilla (CaS1, CaS2) (Figs. 1 and 2). The TS are distinguished by medium–long, straight or slightly curved thin bristles characterized by a smooth wall with a sharp tip (Figs. 1B, E, 2A, C, F). The ChS are bristles characterized by a flexible circular membrane at the base and marked longitudinally by arranging grooves (Figs. 1B–E, 2B, C). The ChS3 is the longest one, followed by ChS1, whereas ChS2 is the shortest sensillum of the chaeticum type. The BS1 is a short and thick peg with an inflexible socket, characterized by a porous wall and uniform thickness with a distinctive blunt tip that has a typical cone shape (Figs. 1D, E, 2D, E). The BS2 is a very short, smooth-walled, cone-shaped sensillum distinguished by a widened and bifurcated tip (Fig. 2G–I). Two subtypes of CaS are found on the last two flagellomeres by two morphological appearances. Groups of aporous CaS1 appear as a smooth elliptical depression on the cuticle with a slightly raised, oval inner area (Fig. 2D, F). However, the other subtype of campaniform sensilla (CaS2) appeared as circular, dome-shaped organ surrounded by a cuticular fold (Fig. 2G).

#### Distribution and size of sensilla

The scape has four to six TS. Their lengths range from 25.45 to 33.37 µm. The pedicel bears two types of sensilla: TS and ChS subtype 1. No ChS2 were found on the pedicel. The ChS1 are the most abundant sensilla type (29–32) on the pedicel segment, with a mean length of 29.54 ± 1.87 μm (Fig. 1B). The TS (14–16 in number) are slightly shorter than the ChS1, with a mean length of 27.25 ± 2.44 μm.

The first seven flagellomeres carry various types of sensilla: TS, ChS subtype 1, and ChS subtype 2 (Fig. 1C). The TS (seven to nine per flagellomere) have a mean length of 30.76 ± 2.48 μm. The ChS1 and ChS2 are significantly different in length (Fig. 2A–C). The ChS1 are the most abundant sensilla type (32–36 in number), distributed on all flagellomeres and characterized by upright, slightly curved, and grooved wall bristles. The lengths of ChS1 range between 32.34 and 40.30 µm, with a mean length of 36.87 ± 2.54 µm. ChS1 are longer than ChS2 (14.02 ± 1.26 µm). The ChS2 are fewer (six to nine) per flagellomere than ChS1 for the first seven flagellomeres. The terminal flagellomere bears eight sensillum types (TS, three subtypes of ChS (1, 2, 3), two subtypes of BS (1, 2), and two subtypes of CaS (1, 2)) (Figs. 1D, E, and 2A–I). Fewer (two to three) TS were found on the terminal flagellomere than on other flagellomeres (six to nine), with a mean length of 23.51 ± 1.76 µm. Similarly, fourteen ChS1 were counted on the terminal flagellomere, with a mean length of 35.02 ± 4.56 µm, whereas four to five ChS2 were found on the terminal flagellomere, with a mean length of 16.67 ± 2.93 µm (Fig. 1D, E). A pair of large and sharp ChS (bristle-shape) with a grooved wall (ChS subtype 3) was found on the apical tip of the terminal flagellomere (Fig. 1D, E). The ChS3 on the terminal flagellomere are the longest and broadest ChS type on the antennal segments of male *P. solenopsis*. The mean length of ChS3 is 41.70 ± 0.79 µm, with a diameter of 3.00 ± 0.16 µm. The thickness of ChS3 is twice that of ChS1 (1.5 µm) as shown in Fig. 2G. Additionally, a basiconic sensillum subtype 1 (BS1) is found on the last two flagellar segments (seventh and eighth flagellomeres) as shown in Figs. 1D, E, 2D, E. On the last two flagellomeres, one and two BS1 were present on the seventh and the eighth flagellomere segment, respectively. For the eighth flagellomere, BS1 were present on the midline of the flagellomere, with a mean length of 15.18 µm (Figs. 1E and 2G). The BS subtype 2 was found only as a unique sensillum at the tip of the eighth flagellomere (Fig. 2G–I) and appeared extremely short in length (2.10 µm). The campaniform sensilla are also present on the last two flagellomeres (seventh and eighth) as shown in Fig. 2D–I. The CaS shows two morphological appearances (CaS subtype 1 and CaS subtype 2). Groups of aporous CaS1 are found on the base of the cuticle located among different sensillum types (Figs. 2D–F, H, I). Moreover, a slightly larger CaS1 is found at the extreme tip of the terminal flagellomere near BS2 (Fig. 2H, I). However, the second subtype (CaS2) was found only as a unique sensillum on the distal end of the terminal flagellomere (Fig. 2G).

### Female antennae

#### Antennal morphology

**Fig. 3:**
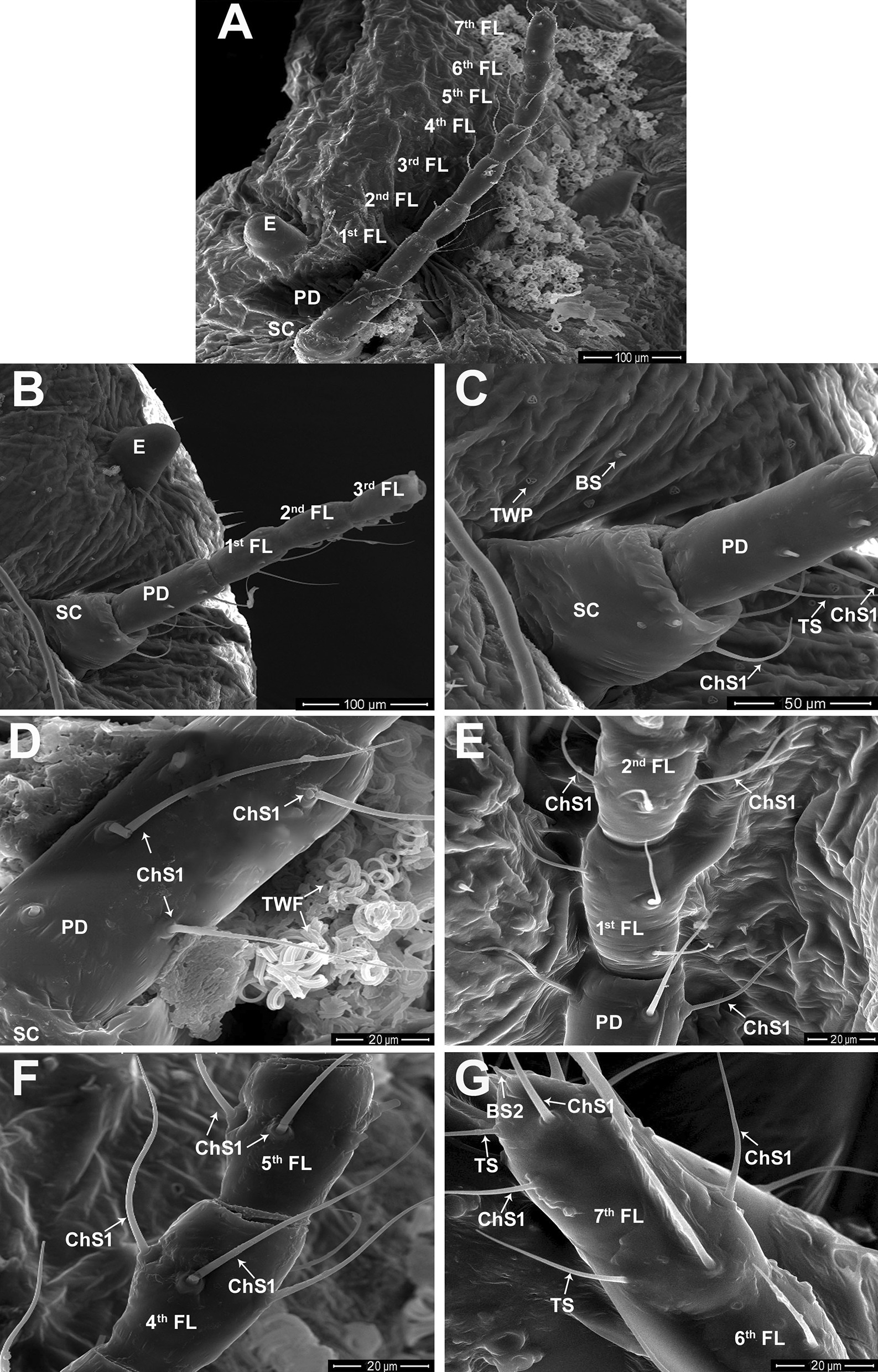
Scanning electron microscopy (SEM) micrographs of the female antenna of *P. solenopsis*. **A** The eye (E) and different antennal segments: the scape (SC), pedicel (PD), and flagellum consisting of seven flagellomeres (FL). **B** The SC, PD, and the first to third flagellar segments. **C** A magnified view of SC and PD segments bears chaetica sensilla type 1 (ChS1). Part of cuticle associated with various trilocular wax pores (TWP), and short basiconic sensilla (BS). **D** A magnified view of the PD segment bears ChS1, and a bunch of trilocular wax filaments (TWF). **E** A part of antennal segments: PD, first, and second flagellomeres carry ChS1. **F** Two flagellar segments (fourth and fifth flagellomeres) carries chaetica sensilla subtype 1 (ChS1). **G** A part of the sixth flagellomere and seventh flagellomere associated with ChS1, TS, and BS2 sensilla

**Fig. 4:**
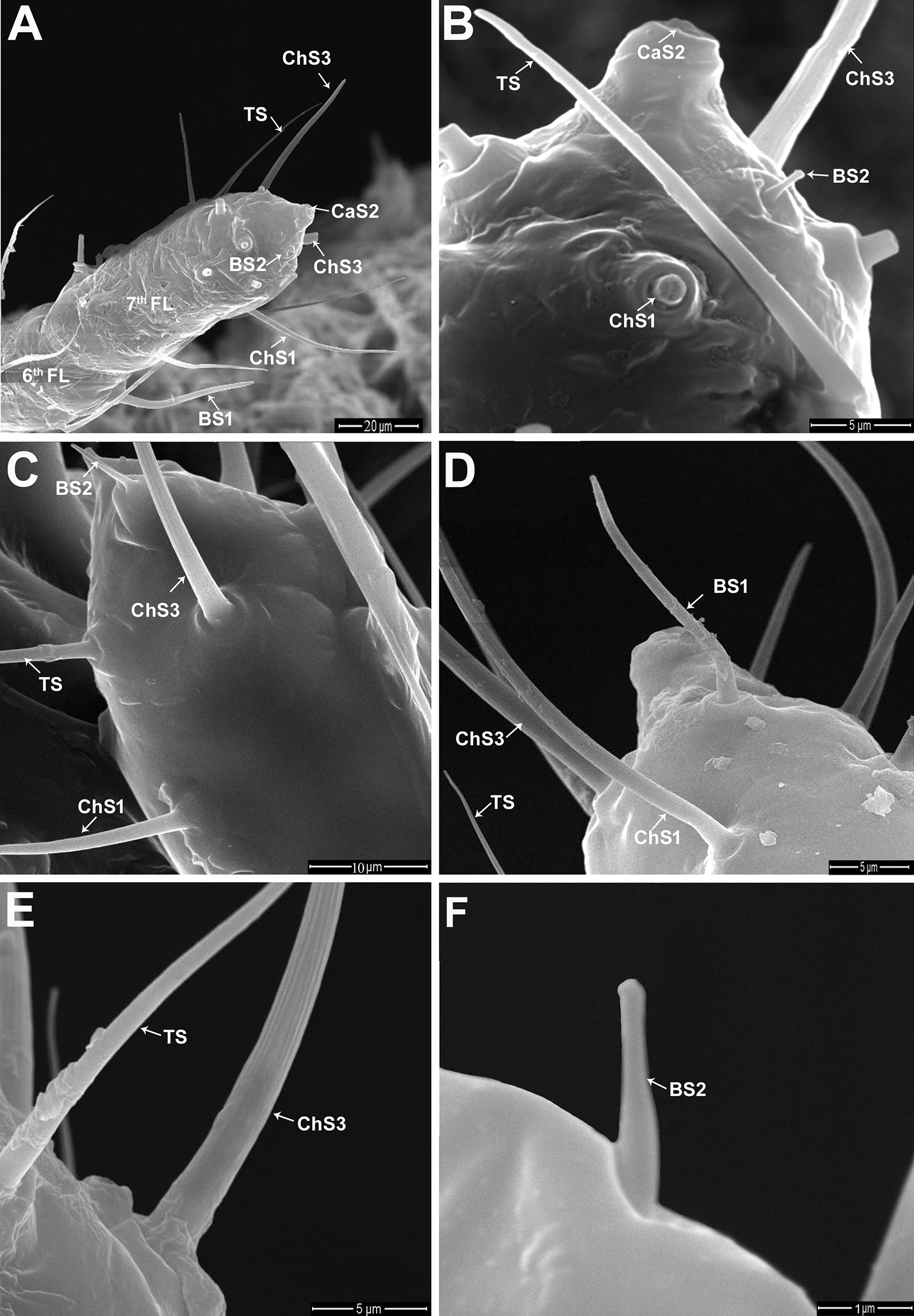
Scanning electron microscopy (SEM) micrographs of the female antenna of *P. solenopsis*. **A** A part of the sixth flagellomere carries sensillum basiconicum subtype 1 (BS1); moreover, the seventh flagellomere bears chaetica sensilla subtype 1 (ChS1), a broken ChS subtype 3 (ChS3), TS, BS subtype 2 (BS2), and CaS subtybe 2 (CaS2). **B** Magnified view of the tip of the seventh flagellomere is associated with a broken ChS1, ChS3, TS, CaS2, and BS2. **C** Magnified view for the terminal flagellar segment showing ChS1, ChS3, TS, and an expanded BS2 sensillum. **D** Magnified view for the terminal flagellar segment showing ChS1, ChS3, TS, and BS1. **E** Higher magnifications for ChS3 and smooth-walled TS sensillum. **F** Higher magnification for BS2 as a short smooth-walled peg

The female antennae consist of nine segments grouped into three regions: scape, pedicel, and flagellum (Fig. 3A). The entire length of the female antenna is 546.04 ± 9.20 µm (mean ± S.D.). The antennal segments are illustrated in Figs. 3 and 4. The scape appears as a short segment with a mean length of 58.24 ± 2.56 μm and is the broadest antennal segment (65.08 ± 3.38 μm wide). The pedicel is a slender structure and the longest antennal segment with a mean length of 90.67 ± 2.75 μm and a width of 37.79 ± 1.76 μm (Fig. 3B, C). The flagellum consists of seven flagellomeres with lengths varying between 38.66 and 77.14 μm. The seventh flagellomere is the longest segment, with a mean length of 76.39 ± 2.07 μm and a width of 29.16 ± 1.32 μm. The first flagellomere is the broadest flagellum segment, with a width of 32.57 ± 1.45 μm, and the second longest flagellomere, with a mean length of 65.46 ± 5.91 μm. The sixth flagellomere is the smallest antennal segment (38.66 ± 2.06 µm in length and 27.22 ± 0.74 µm in width), followed by the fifth flagellomere, with a mean length of 48.86 ± 2.76 μm and a width of 28.35 ± 1.06 μm. The third flagellomere is the third longest, with a mean length of 61.98 ± 6.96 μm and a width of 28.33 ± 1.55 μm. The second and fourth flagellomeres have intermediate size, with mean lengths of 58.26 ± 2.06 μm and 52.22 ± 3.19 μm and widths of 31.03 ± 2.20 μm and 28.94 ± 1.22 μm, respectively.

#### Types of female sensilla

Two subtypes of sensilla chaetica (ChS1 and ChS3), two subtypes of basiconic sensilla (BS1 and BS2), trichoid sensilla (TS), and sensillum campaniformium subtype 2 (CaS2) were identified on different antennal segments of *P. solenopsis* females (Figs. 3 and 4). The ChS1 are distinguished as upright bristles with slightly curved and grooved walls and flexible circular membranes at the base (Fig. 3D–G). The wax pores (TWP) and filaments (TWF) of *P. solenopsis* were illustrated in Fig. 3C, D respectively. These hydrophobic wax filaments are secreted by various dermal pores upon invasion by microorganisms and parasites. The TWF serve as a protectant, preventing physical and chemical damage at various developmental stages. A pair of ChS3 appears only on the terminal flagellomere as sharp bristle-shaped sensilla with longitudinally arranged grooves (Fig. 4A–E). The TS sensilla are distinguished by straight thin bristles characterized by a smooth wall with a sharp tip (Fig. 4A, B, E). The BS1 are characterized by a thick wall, with a flexible socket and a distinctive blunt tip (Fig. 4A, D). By contrast, the BS2 were appeared as a short smooth peg characterized by a conical shape and a rigid socket (Fig. 4B, C, F). The CaS2 were appeared as a circular, dome-shaped organs surrounded by a cuticular fold (Fig. 4A, B).

#### Distribution and size of sensilla

The ChS1 are the most abundant sensillum type on all antennal segments in females, with lengths ranging between 31.35 and 60.54 µm. The scape has three to four ChS1 that are distributed on the tip of the segment, with a mean length of 40.56 ± 3.01 µm (Fig. 3B, C). The pedicel bears eight ChS1 distributed throughout the segment surface, with a mean length of 54.14 ± 3.74 µm (Fig. 3D). The first five flagellomeres of the flagellum carry ChS1. Seven to nine ChS1 are present on the first flagellomere and fewer number (five to six) on other flagellomeres except the terminal one (Fig. 3E–G). Four to five ChS1 are present on the sixth flagellomere and 10–12 on the terminal seventh flagellomere (Fig. 4G). The ChS1 are differentially distributed among the flagellomeres. On the first flagellomere, ChS1 are distributed on the base and top of the flagellomere (Fig. 3A, E), whereas it is distributed in the midline of the second to sixth flagellar segments (Fig. 3A, G).

Unlike the male antennae, the ChS2 are not found on the female antennae. The ChS3 and TS are only found on the seventh flagellomere (Fig. 4A–E). The ChS3 are slightly shorter than ChS1 (35.10 ± 0.86 µm in length) as shown in Fig. 4A. The BS1 are only found on the last two terminal flagellomeres (Fig. 4A, D). Similar to the male antennae, a unique BS2 is observed at the tip of the terminal flagellomere in female antennae (Fig. 4A–C, F). The mean lengths of the BS1 and BS2 are 35.01 ± 0.73 µm and 3.71 ± 1.11 µm, respectively. One CaS2 is found on the distal end of the seventh flagellomere (Fig. 4B).

## Discussion

Few studies have detailed sensilla type and distribution in a mealybug antenna. Salama [30], Koteja [31], Le Rü et al*.* [26], and Karam et al. [29] identified various types of sensilla on the female antenna of the citrus mealybug *Pl. citri* Risso, *P. manihoti,* and *F. malvastra*. To our knowledge, this is the first report to identify the type and distribution of sensilla on the antenna of adult male and female *P. solenopsis.* Detailed descriptions of sensillum types in the antennae of male are lacking for any mealybug species. To our knowledge, this study is the first on the identification of sensillum types in the male mealybug antenna. The male antenna consists of 10 segments (scape, pedicel, and eight flagellar segments). The pedicel of the male *P. solenopsis* antenna is the broadest antennal segment (Fig. 1A). The fourth flagellomere is the longest antennal segment, probably because it functions as a central support axis for flexible antennal movement during mechanical contact with the female body. This point of view is reinforced by data on the thickness of the subsequent antennal segments (fifth to seventh), which are 20%–22% thinner making them fragile and breakable if a central axis is absent.

In this study, we identified eight and six types of sensilla on the antennae of male and female *P. solenopsis*, respectively. In the antennae of adult males, we identified trichoid sensilla, three subtypes of chaetic sensilla, two subtypes of basiconic sensilla, and two subtypes of campaniform sensilla. We identified two subtypes of ChS, two subtypes of BS, TS, and CaS subtype 2 on the antennae of adult females. The identity and distribution of different sensilla types on the antennae of male and female insects may help elucidate the functional roles of these sensilla in mating and feeding behavior. For example, in the present study CaS1 and ChS2 have been found in all flagellar segments of the male antenna. By contrast, the absence of CaS1 and ChS2 on the female antennae of *P. solenopsis* suggests that these are olfactory chemoreceptive sensilla for detecting female sex pheromones by adult males. Consistently, Koteja [31] reported different types of chaetic sensilla that have been identified as chemoreceptive sensilla in *P. aceris* Signoret. Our results indicate that ChS1 is the most abundant sensillum type distributed in all antennal segments of both male and female *P. solenopsis*. However, the distribution of ChS1 on all antennal segments of adult females, especially the large number (10–12) located on the terminal flagellomere may indicates both gustatory and olfactory functions for these sensillum type. The position of the ChS3 along the male antenna indicates that they may play an essential role in mechano-reception during mating. The ChS3 of the adult male could help in identifying the physical characteristics of the female. However, the presence of the ChS3 on the terminal flagellomere of adult females of *P. solenopsis* suggests that the ChS3 play a role in tactile chemoreception and physical communication with the plant surface to gather information about the food source. McIver [32] and Le Rü et al. [26, 27] reported that TS might have an exclusively mechanoreceptive function on the antenna of adult females of *P. manihoti*. In the present study, the CaS2 were reported on the seventh flagellomere of the male antenna and on the terminal flagellomere of *P. solenopsis* female. The CaS have been found wherever the extent of flexion at the joints occurs. Gnatzy et al. suggested that CaS acts as a mechanosensilla activated when the surrounding cuticle is distorted by mechanical stress [33]. For female of *P. solenopsis*, the function of CaS2 may be attributed to monitor the position of the antenna towards the food source that agreed with Abd El-Ghany and Faucheux [34]. They reported that the CaS2 is proprioceptor that stimulated when the pointed tip of the antennae is pressed against the host-plant.

The present study indicates BS1 on the last two flagellar segments and one BS2 on the terminal flagellomere of both sexes of *P. solenopsis*. Le Rü et al*.* [26] suggested that BS1 act as mechanoreceptors and chemoreceptors. However, Altner et al*.* [35] and Le Rü et al*.* [26] suggested that BS2 acts as a thermo-hygroreceptor in *P. manihoti*. They suggested its function due to their low number and distribution on the tip of the antennae in both sexes as a short-blunt peg-like shape with inflexible sockets. In an early study on *Pl. citri*, BS were reported to be present on the subapical and apical antennal segments as olfactory receptors [30]. Similarly, Koteja [31] suggested that the BS found on the apical antennal segments of *P. aceris* may have an olfactory function*.*

A few studies have been conducted on the morphology and ultrastructural characteristics of the antennae in female mealybugs of some species [26, 28, 29]. Sirisena et al. [28] compared the number of antennal segments between *P. solenopsis* female adults with those females of five mealybug species: *C. insolita*, *D. brevipes*, *D. neobrevipes, M. hirsutus*, and *Pl. lilacinus*. Nine antennal segments were identified for *C. insolita, M. hirsutus,* and *P. solenopsis* and eight in *D. brevipes, D. neobrevipes,* and *Pl. lilacinus*. The results for the number of antennal segments are consistent with those of Sirisena et al. [28] for *P. solenopsis* female adults and Karam et al*.* [29] for *F. malvastra*. The scape is the broadest antennal segment in the antennae female of *P. solenopsis*. This finding is consistent with that of Karam et al*.* [29] for the antennae of female *F. malvastra.* Our results indicate that the pedicel is the longest antennal segment in the adult female of *P. solenopsis*. By contrast, the terminal flagellomere is the longest according to Sirisena et al. [28] in the female antennae of six mealybug species and according to Karam et al*.* [29] for *F. malvastra*.

Interestingly, sensillum types and distribution, and thus, potential functions, vary among mealybug species. For the female antenna of *P. manihoti,* different types of sensilla were observed: trichoid, campaniform, coeloconic, and six subtypes of basiconic sensilla (short conical peg; long smooth pegs of types 1, 2, 3; and long grooved pegs of types 4 and 5) [26]. Our findings indicate the absence of coeloconic sensilla for the antennae of female *P. solenopsis*. Moreover, ChS subtype 1 were distributed on all antennal segments of female *P. solenopsis* unlike what reported for the female antenna of *P. manihoti* [26]. Le Rü et al*.* [26] suggested that BS act as mechanoreceptors and chemoreceptors. According to Altner [36] and Zacharuk [37], the sensilla located on the last three flagellar segments are gustatory and olfactory chemoreceptive sensilla. Koteja [31] described the BS and ChS presented on the last antennal segment of the female antennae as sensory receptors in a few species of Pseudococcidae.

## Conclusions

We investigated the olfactory sensilla in the antennae of male and female *P. solenopsis* using SEM. Insect antennae are an invaluable model for studying the fundamentals of odor perception. Results indicate variations in sensillum types and distribution in the antennal segments of male and female *P. solenopsis*. Our findings provide insights for electrophysiological and behavioral studies on chemical communication in insects, particularly the cotton mealybug, *P. solenopsis*, thereby providing a theoretical basis for the development of specific control strategies for *P. solenopsis* in integrated pest management programs.

## Methods

### Insect rearing

The adult females of *P. solenopsis* were reared on sprouting potato tubers under laboratory conditions: 26 ± 2 °C temperature, 60–70% relative humidity, and 16 h light–8 h dark photoperiod. Newly hatched crawlers were placed on each sprouted potato plant and then confined to a cylindrical box of glass, with a length of 25 cm and a diameter of 8 cm. One to 2 days after emergence, males and females were collected for SEM.

### Morphological observation via SEM

We used SEM to investigate sensilla distribution in the antennae of male and female insects. Twenty-five adults of each sex were collected from the laboratory colony and stored in 70% ethanol. The insects were gradually dehydrated using a series of ethanol concentrations (80%, 90%, 95%, and 100% [v/v]) to avoid distorting the samples. The waxy layer that affects the investigation process of fine structures, such as sensilla, was removed using a modified method by Sirisena et al. [28] by soaking the samples for 10 min with hexane instead of chloroform [38]. Finally, the samples were rinsed in 100% ethanol to ensure complete removal of water, and oriented and mounted on aluminum stubs with double-sided sticky tape. The samples were sputter-coated with the carbon coating film, using the High-Resolution Turbomolecular-pumped coater system (Q150T ES, Quorum Technologies Ltd., United Kingdom). The samples were photographed using SEM (Model TESCAN VEGA3 [thermionic emission SEM system], Tescan, Tescan Orsay Holding, Kohoutovice, Czech Republic).

## Nomenclature and measurement of sensilla

The nomenclature of different sensilla types was performed as described previously by [28, 29, 39]. The number per unit surface area and size of various sensillum type were measured to reflect their distribution on the whole antenna. The sizes (lengths and diameters) of various sensilla types were measured using the ImageJ software (http://imagej.nih.gov/ij).

## Data Availability

All data generated or analyzed during this study are included in this published article [and its supplementary information files].
